# Preserved gait kinematics during controlled body unloading

**DOI:** 10.1186/s12984-017-0239-9

**Published:** 2017-04-04

**Authors:** L. Awai, M. Franz, C. S. Easthope, H. Vallery, A. Curt, M. Bolliger

**Affiliations:** 1 Spinal Cord Injury Center, Balgrist University Hospital, Forchstrasse 340, 8008 Zurich, Switzerland; 2 Sobell Department of Motor Neuroscience and Movement Disorders, UCL Institute of Neurology, University College London, 33 Queen Square, London, WC1N 3BG UK; 3 Department of BioMechanical Engineering, Delft University of Technology, 2628 CD Delft, The Netherlands

**Keywords:** Unloading, Body weight support, Gait pattern, Walking, Healthy

## Abstract

**Background:**

Body weight supported locomotor training was shown to improve walking function in neurological patients and is often performed on a treadmill. However, walking on a treadmill does not mimic natural walking for several reasons: absent self-initiation, less active retraction of leg required and altered afferent input. The superiority of overground training has been suggested in humans and was shown in rats demonstrating greater plasticity especially in descending pathways compared to treadmill training. We therefore developed a body weight support system allowing unrestricted overground walking with minimal interfering forces to train neurological patients. The present study investigated the influence of different amounts of body weight support on gait in healthy individuals.

**Methods:**

Kinematic and electromyographic data of 19 healthy individuals were recorded during overground walking at different levels of body weight support (0, 10, 20, 30, 40, and 50%). Upper body inclination, lower body joint angles and multi-joint coordination as well as time-distance parameters were calculated. Continuous data were analyzed with regard to distinct changes within a gait cycle across all unloading conditions.

**Results:**

Temporal gait parameters were most sensitive to changes in body unloading while spatial variables (step length, joint angles) showed modest responses when unloaded by as much as 50% body weight. The activation of the gastrocnemius muscle showed a gradual decrease with increasing unloading while the biceps femoris muscle showed increased activity levels at 50% unloading. These changes occurred during stance phase while swing phase activity remained unaltered.

**Conclusions:**

Healthy individuals were able to keep their walking kinematics strikingly constant even when unloaded by half of their body weight, suggesting that the weight support system permits a physiological gait pattern. However, maintaining a given walking speed using close-to-normal kinematics while being unloaded was achieved by adapting muscle activity patterns. Interestingly, the required propulsion to maintain speed was not achieved by means of increased gastrocnemius activity at push-off, but rather through elevated biceps femoris activity while retracting the leg during stance phase. It remains to be investigated to what extent neurological patients with gait disorders are able to adapt their gait pattern in response to body unloading.

## Background

In order to improve ambulatory function in subjects with neurological gait disorders such as motor incomplete spinal cord injury, automated locomotor training was shown to be beneficial [1–4] and superior to conventional rehabilitation training [5]. Locomotion is the result of complex interactions of multiple afferent and efferent signals during which a dynamic equilibrium needs to be maintained while coordinating a large number of muscles and body segments. In light of the complexity of this interaction, task specificity and appropriate afferent inputs during training are paramount [6]. In severely affected patients with prominent muscle weakness, partial body weight support (BWS) and sometimes even manual or robotic assistance of leg movements are necessary to enable patients to undergo locomotor training [5]. Most often, for practical and technical reasons, locomotor training is performed on a treadmill. Automated treadmill training bears advantages of providing large amounts of stepping in a confined, controlled environment and therefore also provides a suitable setup for gait analysis. In humans, several studies showed that the functional outcome after rehabilitation did not depend on the type of training (overground, treadmill, functional electrical stimulation) [7–9], while some other studies performed in neurological patients reported beneficial effects of overground training as compared to treadmill walking. Dobkin and colleagues further question the favorable effects of body weight supported treadmill training over overground training [6, 10, 11]. However, these studies investigated changes in ordinal clinical scores or walking speed and distance rather than gait patterns in terms of kinematics and muscle activity. In a study performed in rats, it was shown that locomotor training on a treadmill was less effective at driving neuronal plasticity and recovery of voluntary function as compared to rats that were trained to walk over ground [12]. The authors suggested that the training of voluntary movements (i.e., active overground walking) drives the plasticity of descending pathways, which especially improved skilled walking. In addition, overground locomotor training enables walking on a natural surface including curved walking, turning or obstacle negotiation (e.g., stairs) while providing adequate sensory feedback and requires gait initiation and termination as well as active propulsion of the body in a desired direction. All these features are essential for ambulation in everyday life and are difficult to train on a treadmill. Overground training is, however, challenging because neurological disorders often entail muscle weakness and motor control deficits that impede natural walking and create risks of falling.

In recent years, BWS systems that enable overground walking have been put forward, such as the Zero-G [13], or the NaviGaitor [14]. In the design of these systems, it is critical to minimize interaction forces, as these might alter gait dynamics. Minimal interaction is potentially even more important when such devices are used not for training, but to study gait alterations in neurological patients during natural walking. In all these cases, the system should only provide the necessary support for gait training and ensure safety. To achieve this, we introduced a cable-based BWS system that enables three-dimensional overground walking in humans, while at the same time minimizing unwanted interaction forces [15]. The robotic system FLOAT (LME, Rüdlingen, Switzerland) is an overhead cable system that is designed to precisely control forces acting on a human subject in the vertical and the two horizontal directions [15]. In the FLOAT, these interaction forces compose a single resultant force vector that is transmitted to the person via the harness. This vector is mainly estimated from the elongation of springs that are placed in series with the suspending cables. This design permits various training scenarios (e.g., level walking, stair climbing, sit-to-stand transition) in patients with movement disorders.

The aim of the present study was to characterize alterations in gait patterns induced by different unloading magnitudes using the FLOAT in non-impaired individuals. Previous studies investigating the influence of body unloading during treadmill walking suggested changes in gait phase timing, inconsistent changes in kinematic parameters and significant alterations in distinct lower limb muscle activation patterns [16, 17]. One study exploring gait changes induced by an overground support system found significant kinematic [18] and electromyographic (EMG) alterations [19] in unimpaired overground walking, although they did not investigate muscles that contribute to leg retraction. Understanding alterations of gait characteristics under the influence of a BWS system in healthy individuals is a prerequisite for a contextual interpretation of gait behavior in subjects recovering from neurological conditions who train with comparable rehabilitative devices. The findings may help to tailor the most suitable training program to the specific condition of individual patients.

## Methods

### Participants

19 healthy volunteers (9 females and 10 males, age 29 ± 5 years (mean ± 1SD), height: 1.74 ± 0.09 m, weight: 72 ± 12 kg) participated in this study and gave their written informed consent. The study was approved by the local ethics committee of the Canton of Zurich and was conducted in accordance with the Declaration of Helsinki.

### Materials

**Fig. 1:**
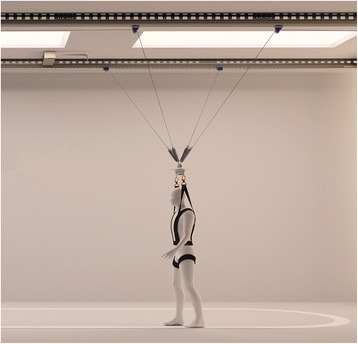
The cable robot is powered by four motors that actuate a central node via a ceiling-mounted rail- and deflector system. The design of the body weight support system allows unrestricted walking on level ground or on stairs as well as training paradigms such as sit-to-stand transition

A BWS system (The FLOAT, LME, Rüdlingen, Switzerland) unloaded participants during walking over ground (Fig. 1). The cable robot was powered by four motors actuating a central node via a rail- and deflector system. The subjects wore a harness that was attached to the node. Force sensors mounted between the node and the cables controlled the unloading force, which was commanded to be purely vertical. This setup allowed subjects to walk freely in an area of approximately 8 x 2 m. A detailed description of the system can be found elsewhere [15]. Gait kinematics were recorded using an optical 3D motion tracking system at 200 Hz sampling frequency (Vicon motion systems Ltd., Oxford, UK). EMG activity was recorded at 1500 Hz using a wireless EMG system (Noraxon Inc., Arizona, USA) with dual surface electrodes placed over the following lower limb muscles: rectus femoris (RF), biceps femoris (BF), tibialis anterior (TA), and gastrocnemius medialis (GM).

### Setup and protocol

Subjects walked barefooted over ground along an 8 m walkway, while only the middle ~6 m were analyzed in order to exclude acceleration and deceleration phases. We recorded at least 20 complete gait cycles per condition, which resulted in approximately 6 trials per condition in most subjects. The subjects were instructed to walk at a given walking speed (0.56 m/s). This rather slow walking speed was chosen in order to match walking speeds observed in neurological patients for whom the BWS system was designed. The walking speed was measured by the BWS system and acoustic feedback was presented to the subjects whenever their walking speed was outside a tolerance range (± 0.14 m/s) of the desired speed. Six different unloading conditions were assessed: no unloading (baseline), 10%, 20%, 30%, 40%, and 50% body weight unloading. In the baseline condition, subjects wore the harness and were attached to the device, but the unloading was controlled to be minimal, as necessary to maintain tension in the cables. The order of the 6 conditions was pseudorandomized.

### Data analysis

Data were analyzed offline. Per subject and condition 20 gait cycles (from heel strike to heel strike) were analyzed. Kinematic data was acquired and post-processed using Vicon Nexus Software (1.7.1 and 1.8.3). The processing included reconstruction of data points, filling of trajectory gaps and smoothing of trajectories using Woltring’s cross-validatory quintic-spline routine with a mean squared error of 10 mm^2^. Joint angles were calculated from the Vicon Nexus Plug-in Gait full body model (v 3.0). Further kinematic data analysis was performed using custom-written MATLAB scripts (The Mathworks Inc., Natick, USA). All continuous data (i.e., kinematic and EMG data) was cut into individual gait cycles (from heel strike of one foot to the consecutive heel strike of the same foot identified using the trajectory of the heel marker) and time-normalized so that the stance- and swing phases had the same relative lengths across all unloading conditions (defined by the mean time point of toe-off over all conditions).

### Outcome measures

The following time-distance parameters were derived from the kinematic data: step length, cadence, stance phase, swing phase, single support phase, and double support phase. The upper body inclination angle was calculated as the angle between the vector pointing from the center of the pelvis segment towards the center of the thorax segment and the earth-vertical axis, whereby all vectors were taken in the sagittal plane. Positive values indicate a forward tilt while negative values refer to a backward tilt. From the recorded joint trajectories, joint angles at heel strike and toe-off as well as the ranges of motion of hip, knee and ankle joints (calculated as the difference between minimal and maximal deflection angles during a gait cycle) were extracted. The temporal profiles of two adjacent joint angles were conducted via so-called cyclograms. Cyclograms contain information on interjoint coordination and exhibit a characteristic shape under specific conditions (e.g., walking speed), which had previously been shown to be strikingly similar between healthy individuals walking at their comfortable walking speed, while showing distinct alterations in a neurological population [20]. Some features of the cyclogram (cycle-to-cycle consistency and shape normality) that were shown to be sensitive readouts for motor control deficits [20, 21] were quantified by the angular component of coefficient of correspondence (ACC) and the square root of the sum of squared distances (SSD; for details see [22] and [20]), which represents the cyclogram shape deviation from a norm cyclogram (in this case baseline walking with no unloading). This measure can be interpreted as a metric for the quality of interjoint coordination [20].

The EMG signal was offset corrected, filtered using a recursive fifth-order Butterworth bandpass filter (10–500 Hz), rectified and smoothed using a moving average filter with a window width of 11 prior to statistical analysis. The EMG amplitudes were normalized to the mean of the uppermost 5% of EMG activity during baseline condition for each person. Continuous EMG data was then cut into stance phase and swing phase, time-normalized to the mean of stance phase and swing phase over all conditions (i.e., 625 and 375 samples, respectively) and subsequently concatenated to form a 1000 sample trajectory representing an entire gait cycle.

### Statistical analysis

For statistical analysis, the mean values of the left and right legs were evaluated. Statistical comparisons of time-distance parameters and interjoint coordination measures across the different unloading conditions were performed using a repeated measures General Linear Model (rmGLM), while post-hoc pairwise comparisons were carried out using paired t-tests between each unloading condition and baseline (0% BWS). Multiple testing was accounted for by applying a Sidak correction. If the assumption of sphericity was violated (Mauchly’s Test), the corresponding *p*-values were corrected by applying a Greenhouse-Geisser correction. These statistical tests were carried out using IBM SPSS Statistics 22 (IBM Corp., Armonk, NY, USA). EMG and kinematic time-series data were analyzed using statistical parametric mapping [23] using Matlab (R2015a) in a one-way ANOVA design for unloading condition [24]. Critical thresholds were defined by generating random fields representative of the recorded data in smoothness and amplitude and adopting a threshold value ensuring that 95% of the random fields remained within these bounds (alpha = 0.05) [24, 25]. Post-hoc pairwise comparisons were performed between baseline and each unloading level using a Sidak correction when the critical threshold was exceeded during the first-level comparison to retain a Type I family-wise error rate of alpha = 0.05. Time periods during which the trajectories were significantly different were retained and the probability reported for the occurrence of all clusters. Use of this methodology enabled the detection of significantly different sections within the time trajectories.

## Results

### Time-distance parameters

Time-distance parameters showed a broad range of changes with respect to body unloading. Step length was significantly increased at 30% BWS compared to baseline and cadence was reduced at 40% and 50% BWS. Stance phase and double support phase were significantly reduced at 20% through 50% BWS hence single support- and swing phase were prolonged at these respective unloading conditions (see Table 1 for a summary of results from the rmGLM and pairwise post-hoc comparisons).

**Table 1:** Contrasts of different levels of unloading compared to baseline

Parameter	BL	10%	20%	30%	40%	50%
Step length [m]*	0.435 (0.036)	0.438 (0.043)	0.444 (0.045)	**0.457 (0.048)**	0.445 (0.046)	0.463 (0.051)
Cadence [steps/min]**	80.75 (6.38)	80.66 (7.88)	79.46 (8.02)	78.13 (8.89)	**77.17 (9.10)**	**74.23 (9.64)**
Stance phase [%]**	64.41 (1.09)	64.19 (1.16)	***63.21 (1.33)***	***62.23 (1.60)***	***61.14 (1.77)***	***59.42 (2.19)***
Swing phase [%]**	35.59 (1.09)	35.81 (1.16)	***36.79 (1.33)***	***37.77 (1.60)***	***38.86 (1.77)***	***40.58 (2.19)***
Single support phase [%]**	35.62 (1.05)	35.75 (1.17)	***36.76 (1.34)***	***37.90 (1.82)***	***38.93 (1.78)***	***40.48 (2.20)***
Double support phase [%]**	28.79 (2.13)	28.45 (2.32)	***26.45 (2.67)***	***24.33 (3.38)***	***22.20 (3.54)***	***18.94 (4.32)***
ACC hip-knee**	0.761	0.791	0.753	0.716	0.788	0.818
ACC knee-ankle**	0.744	0.773	0.732	0.695	0.759	0.778
SSD hip-knee [a.u.]**	0	***2.07***	***3.41***	***5.39***	***7.95***	***11.34***
SSD knee-ankle [a.u.]**	0	***1.58***	***3.37***	***5.37***	***8.21***	***11.26***

The outcome of the repeated measures general linear model (rmGLM) is represented by asterisks: * = *p* < 0.05 and ** = *p* < 0.01. Mean values (standard deviation in brackets) for each level of unloading and baseline are displayed on the right. Values highlighted in bold reflect *p*-values < 0.05 resulting from post-hoc paired t-tests and values highlighted in bold and italic represent *p*-values < 0.01. Percentages indicate % of gait cycle. ACC = angular component of coefficient of correspondence, a.u. = arbitrary units, SSD = square root of sum of squared distances

### Gait kinematics

**Fig. 2:**
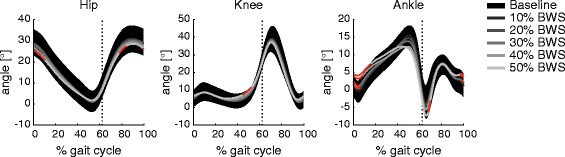
Angular trajectories of the hip-, knee-, and ankle angle in the sagittal plane during a gait cycle are shown across all levels of unloading (black area shows the ± 1 standard deviation interval during baseline condition). The time intervals during which significant differences between unloaded condition and baseline were found are highlighted in red. The vertical line indicates the mean time point of toe-off across all levels of unloading. BWS = body weight support

**Fig. 3:**
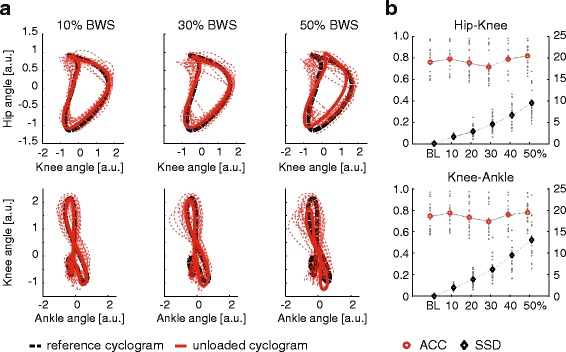
**a** The hip-knee and knee-ankle cyclograms at 10, 30, and 50% body weight support (BWS) are depicted in reference to the baseline condition (thick black dashed line). The thick red line represents the mean value across all subjects while the thin red dotted lines are individual trajectories. The cyclograms were normalized to have unit size (1) that allowed the calculation of the shape difference. **b** The values for the cycle-to-cycle consistency (ACC) and shape deviation (SSD) are shown on the right. ACC = angular component of coefficient of correspondence, a.u. = arbitrary units, BWS = body weight support, SSD = square root of sum of squared distances

The time trajectories of the hip- and knee angles during a gait cycle varied little between the unloading conditions (Fig. 2), which was reflected by the trajectories remaining within the interval of ±1SD for all conditions. Only the ankle angle consistently showed significant differences in the different unloading conditions that was also outside of the ±1SD range of baseline walking: with increasing amounts of unloading the ankle showed more dorsiflexion at heel strike (and throughout the initial part of stance phase, Fig. 2). The rmGLM suggested an effect of body unloading on cyclogram consistency (ACC) for both the proximal hip-knee coordination (rmGLM: F = 8.22, *p* < 0.001) and the distal joints (knee-ankle; rmGLM: F = 5.97, *p* < 0.001). However, post-hoc pairwise comparisons did not yield any significant differences in the consistency of interjoint coordination between the various unloading conditions with respect to baseline (Table 1). By contrast, the shape difference to baseline of the cyclogram and thus the quality of interjoint coordination changed with body unloading (Fig. 3, Table 1). This was true for both hip-knee coordination (rmGLM: F = 54.12, *p* < 0.001) as well as the knee-ankle cyclogram (rmGLM: F = 43.67, *p* < 0.001).

**Fig. 4:**
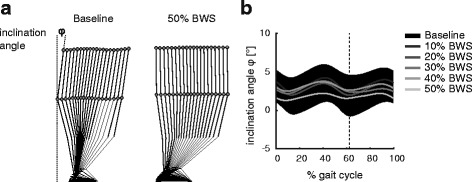
**a** The stickfigures of one subject are shown during baseline walking and at 50% body weight support (BWS). The inclination angle φ was calculated as the angle between the thorax – pelvis vector in the sagittal plane and earth-vertical axis and is shown for the entire gait cycle **b.** The inclination angle during a gait cycle across all levels of unloading is shown (*black area* shows the ± 1 standard deviation interval during baseline condition) and the vertical dashed line indicates the mean time point of toe-off across all levels of unloading

The upper body inclination angle seemed to decrease with increasing levels of body weight unloading, although it did not reach statistical significance. In other words, the higher the BWS, the more upright the posture tended to become (Fig. 4).

### Muscle activity

**Fig. 5:**
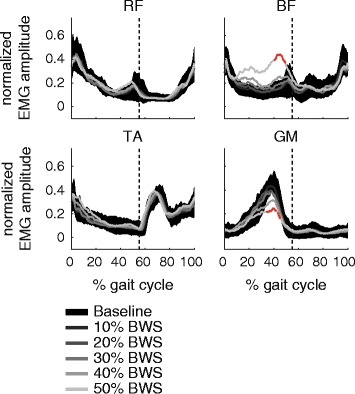
The averaged EMG traces are shown for all levels of unloading (*black area* shows the ± 1 standard deviation interval during baseline condition). The time intervals during which significant differences between unloaded condition and baseline were found are highlighted in *red*. The *vertical dashed line* indicates the time point of toe-off. BF = biceps femoris, BWS = body weight support, EMG = electromyogram, GM = gastrocnemius medialis, RF = rectus femoris, TA = tibialis anterior

One subject was excluded from the EMG analysis because of technical problems during the acquisition. Among the other subjects, neither RF nor TA showed statistically significant alterations (spm1d: *p* > 0.05) induced by body unloading (Fig. 5). By contrast, BF and GM showed some effects with respect to unloading condition (spm1d: F = 4.42, *p* < 0.001 for BF and F = 4.28, *p* < 0.001 for GM). Post-hoc tests revealed that BF showed an increased activity level at 50% BWS during stance phase (spm1d: t = 4.85, *p* < 0.01), while the GM muscle yielded diminished amplitudes at 50% BWS during 3 time intervals within stance phase (spm1d: t = 4.99, *p* < 0.001 for all 3 clusters). None of the muscles showed any alterations during swing phase.

## Discussion

The present study investigated the influence of minimally obtrusive body-weight support on the gait pattern of healthy individuals walking over ground. For this purpose, participants were unloaded by up to 50% of their body weight while walking at a predefined speed. In summary, albeit profound unloading with abundant effects on time-distance parameters and compensatory alterations in EMG activation, the upper-body posture and single-joint angular trajectories showed well controlled changes that still resembled physiological walking while the complex interjoint coordination of lower limbs showed some adaptations. These findings reveal that the studied BWS system did not profoundly distort gait kinematics even at high amounts of unloading. However, the healthy subjects adjusted gait-phase timing and adopted compensatory muscle activation strategies to preserve lower limb gait kinematics during body unloading while retaining a target speed.

BWS systems are primarily intended to alleviate challenges of weight bearing, thus providing support for patients with neurological deficits and/or muscle weakness to allow remaining neural resources to be directed towards the control of walking. Body unloading inevitably interacts with locomotor control and assessing the kind and extent of adjustments of gait pattern in healthy individuals in response to changing environments will provide insights into the capacity of human gait control. Knowledge of the latter is a prerequisite for the evaluation of gait alterations during self-initiated overground walking in neurological patients.

### Effects of unloading on motor control

Human walking and gait control have been studied extensively in the past both in animals and humans and several emerging concepts are widely accepted. In humans, the anatomy and function of neural pathways were often examined using electrophysiological methods. Reflexes allow the study of both sensory and motor pathways with peripheral and central components. Behavior of reflex responses may provide insight into the function and state of the sensory-motor system and how it is affected by different conditions. One of these studies revealed that cutaneous reflex responses were generally enhanced by body unloading [26], similar to exaggerated reflex amplitudes after injuries to the central nervous system, supposedly via diminished supraspinal inhibition of spinal synapses [27, 28]. However, complete unloading during passive walking evoked no modulation of TA muscle reflex responses after tibial or peroneal nerve stimulation [29], suggesting that load-dependent afferent feedback may modulate motor output. The dependence on minimal contact force during walking was also shown in a study by Ivanenko and colleagues [30], where the control of the foot trajectory only deteriorated when no load was applied to the foot, but remained surprisingly stable across unloadings of up to 95%. It is also believed that load-dependent facilitation and inhibition of leg extensor and flexor muscles, respectively, are required to prevent a collapsing of the leg as long as it remains in stance phase [31]. It was shown in both cats [32] and humans [33, 34] that the hip joint plays a crucial role in orchestrating lower limb coordination during the stance to swing transition and it may therefore be presumed that the hip joint underlies more confined control. In support of this assumption, the current findings of a minor proximal-distal imbalance in response to unloading suggest a conserved hip and knee angle trajectory irrespective of unloading while the ankle angle was most responsive. This observation is slightly different from the results obtained during the same amount of body unloading at an identical speed with subjects walking on a treadmill [17]. During treadmill walking (where hip extension is facilitated through the moving belts), the hip showed the greatest deviation from baseline while both knee and ankle angles only deviated from the baseline kinematics at 75% BWS. Similarly, in a recent study looking at the effect of unloading on gait kinematics during overground walking but using a different support system, the effects of body unloading were more abundant in the hip and knee joints as opposed to the ankle [18]. This divergence in outcome may be the consequence of the different weight support systems used. The currently employed cable robot was specifically designed in a way that the system’s masses, which in most cases cause inertial forces to interfere with unimpeded movements, were removed from the subject and therefore prevented inadvertent joint excursions as an attempt to overcome these external forces.

In contrast to the sparsely observed and comparatively minor alterations in single-joint movement trajectories, the interjoint coordination exhibited some susceptibility to decreased body load. Both proximal and distal interjoint coordination (hip-knee and knee-ankle cyclograms) progressively deviated in shape from baseline condition (SSD) with increasing body load, while their cycle-to-cycle reproducibility (ACC) remained unchanged. These findings support previous suggestions that multi-joint coordination is a more sensitive readout for locomotor control and levels of gait impairment as compared to parameters derived from single-joint time trajectories [20, 21]. However, the reported difference in shape is mainly induced through a rotation of the base shape rather than a spatial distortion (as revealed by the robust single-joint trajectories of hip, knee and ankle) suggesting that joints maintain their covariation pattern independent of unloading as has been reported in previous studies [30].

In summary, minor changes in joint kinematics found in the present study suggest that maintaining a normal-appearing walking pattern (i.e., normal interaction with the environment) has a high priority in gait control and therefore kinematic parameters remained largely unchanged even when the body was unloaded by as much as half of the body weight. This may imply that body unloading as it was applied in the present study does not act as a major perturbing factor during normal walking.

### Compensatory muscle activity to preserve walking kinematics

Although minor changes could be seen in most kinematic measures, especially the distal ankle angle, the overall observable walking pattern remained surprisingly unaltered even at high levels of unloading, which is in line with previous research [30]. Maintaining normal kinematics in the presence of reduced ground reaction forces, shear forces and altered afferent feedback is only possible at the expense of an adapted EMG pattern. In order to overcome the decreased interaction forces between the feet and the ground, an increased activation of specific muscle groups during stance phase is required. Sufficient forward acceleration of the center of mass to achieve a given walking speed may be provided via enhanced retraction of the leg during stance phase, an amplified force generation in the ankle extension during push-off, or both. The current data suggests that forward propulsion of the body in light of diminished ground reaction forces was achieved by means of increased activation of the BF during stance phase rather than exaggerated GM muscle activity at push-off, which actually decreased at higher levels of unloading [35]. These results are in line with the adaptations of muscle activity observed during unloading while walking on a treadmill [17, 26, 30]. Although previous work has suggested that extensor muscles are more sensitive to load-related afferences compared to flexor muscles [36], the BF showed greater alterations with body unloading compared to the RF. This may be partly explained by the dual function of the BF both as a knee flexor but also as a hip extensor. In addition, the external conditions imposed on the subjects in the present study (i.e., a given walking speed, reduced interaction forces on the ground, less forward inclination of the upper body) may entail the emerging BF activity during stance phase. The more upright trunk position, for example, may mechanically induce more BF activity as less upper body inclination angle leads to less momentum generated by the upper body during propulsion of the center of mass. However, since the change in upper body inclination angle was minor (did not reach statistical significance), trunk angle might not be the driving force for higher BF activity. While the GM showed gradual decreases in activity level with increasing weight unloading the BF revealed a rather non-linear relationship with changing load afference, supporting the non-linearity of the underlying motor control mechanism reported previously [30]. In summary, investigating the changes in muscle activation patterns invoked by an individual in order to minimize alterations in gait kinematics despite varying external constraints such as body unloading may provide insight into the degree of sensory-motor impairment.

### Implications for patient training

Patients suffering from a stroke or Parkinson’s disease typically show a prolonged stance phase and double support phase while decreasing their swing phase and single support phase compared to healthy control subjects [37, 38]. These alterations are probably the consequence of balance problems in those particular patients, causing them to adopt a more secure and stable gait. Given the results found in the current and previous studies [16–18], body unloading during walking may reverse this ‘maladaptive’ walking pattern, increasing the time spent in unstable gait phases (i.e., swing phase, single support phase) while consequently decreasing stance and double support phases. Thus, a BWS system may contribute to balance training in neurological patients by forcing patients to spend more time in a destabilizing gait phase where they need to actively maintain equilibrium [39].

More extended knee and hip angles at foot-flat [16] and a slightly reduced forward inclination angle (although not significant) during walking found in the current study suggest that an alleviation of body load leads to a more upright body position. In case this translates to spinal cord injured subjects, such an effect might be beneficial in counteracting the crouched posture frequently seen in these subjects. Especially an increased hip extension at the end of stance phase may facilitate the onset of swing phase [33, 34]. However, the underlying reasons for the observed effects are unclear, and they could still be artefacts caused by the harness attachment, or by reduced trunk loading.

### Minor kinematic adaptations and distinct EMG alterations induced by BWS system

Another important outcome of the present study is the finding that gait kinematics changed little as a consequence of unloading using a minimally intrusive BWS system during walking over ground. The main changes in EMG pattern occurred during stance phase while the activation pattern remained fairly unaltered during swing phase. This particular behavior supports the assumption that the BWS system itself, even though constantly attached to the body and acting on its masses, does not per se hamper normal walking beyond the effects of unloading. Otherwise, one would expect abundant alterations irrespective of gait phase. Rather, the reduced load during stance and the ensuing change in afferent signaling (i.e., reduced shear forces, reduced foot sole pressure and diminished joint loads) evoked specific phase-dependent adaptations required to preserve walking kinematics. However, to what extent an intervention of this sort impacts gait in patients with neurological disorders and how that may interfere with the reestablishment of normal walking patterns remains to be investigated and may reveal valuable information on locomotor control after injury. Additionally, future studies may strive to reduce the effects of body unloading by adding supportive forces such as forward pull to facilitate propulsion.

### Limitations

One major drawback of the present study design may be the rather sedate walking speed for unimpaired individuals (0.56 m/s). The reason for this relatively slow speed was the potential comparability of the results to future data obtained from neurological patients. One hallmark of impaired gait in these patients is a limited walking speed.

## Conclusions

Gait phenotype, a result of continuous optimization of motion patterns for energetic efficiency and walking stability, remains remarkably unchanged under body unloading. Specific alterations of muscle activity levels during defined phases of the gait cycle ensure close-to-normal walking kinematics. The fact that the gait pattern was not randomly altered, nor were alterations ubiquitously found, may indicate that the particular design of the studied BWS system did not significantly interfere with normal walking in this cohort of subjects, as this would otherwise have led to general and temporally abundant changes in walking parameters. It remains to be elucidated whether patients abide by similar hierarchical rules of gait control as their unimpaired counterparts and whether their specific deficits actually allow them to adapt to external gait modulation.

## Abbreviations

ACC: Angular component of coefficient of correspondence
BF: Biceps femoris
BWS: Body weight support
EMG: Electromyography
GM: Gastrocnemius medialis
RF: Rectus femoris
rmGLM: Repeated measures general linear model
ROM: Range of motion
spm1d: One-dimensional statistical parametric mapping
SSD: Square root of sum of squared distances
TA: Tibialis anterior
